# Consistent effects of nitrogen fertilization on soil bacterial communities in black soils for two crop seasons in China

**DOI:** 10.1038/s41598-017-03539-6

**Published:** 2017-06-12

**Authors:** Jing Zhou, Xin Jiang, Dan Wei, Baisuo Zhao, Mingchao Ma, Sanfeng Chen, Fengming Cao, Delong Shen, Dawei Guan, Jun Li

**Affiliations:** 1 Institute of Agricultural Resources and Regional Planning, Chinese Academy of Agricultural Sciences, Beijing, 100081 PR China; 2 College of Biological Sciences, China Agricultural University, Beijing, 100094 PR China; 3 The Institute of Soil Fertility and Environmental Sources, Heilongjiang Academy of Agricultural Sciences, Harbin, 150086 PR China; 4 Laboratory of Quality & Safety Risk Assessment for Microbial Products (Beijing), Ministry of Agriculture, Beijing, 100081 PR China

## Abstract

Long-term use of inorganic nitrogen (N) fertilization has greatly influenced the bacterial community in black soil of northeast China. It is unclear how N affects the bacterial community in two successive crop seasons in the same field for this soil type. We sampled soils from a long-term fertilizer experimental field in Harbin city with three N gradients. We applied sequencing and quantitative PCR targeting at the 16S rRNA gene to examine shifts in bacterial communities and test consistent shifts and driving-factors bacterial responses to elevated N additions. N addition decreased soil pH and bacterial 16S rDNA copy numbers, and increased soil N and crop yield. N addition consistently decreased bacterial diversity and altered bacterial community composition, by increasing the relative abundance of Proteobacteria, and decreasing that of Acidobacteria and Nitrospirae in both seasons. Consistent changes in the abundant classes and genera, and the structure of the bacterial communities across both seasons were observed. Our results suggest that increases in N inputs had consistent effects on the richness, diversity and composition of soil bacterial communities across the crop seasons in two continuous years, and the N addition and the subsequent edaphic changes were important factors in shaping bacterial community structures.

## Introduction

The global ecosystem is receiving elevated levels of nitrogen (N), often >100 kg N ha^−1^ y^−1^, in order to achieve high crop yields^1, 2^. The average global rate of N deposition to terrestrial ecosystems is likely to increase by a factor of 2.5 over the next century^3^. Previous studies have shown that elevated N additions to ecosystems lead to climate change, emission of greenhouse gases, species extinction and even human health threats^4^. A significant increase in carbon (C) storage in the form of plant biomass^5^ and productivity^6^, but a loss of plant community diversity^7^ is commonly observed across most experimental N gradients in ecosystems. Furthermore, increased N additions change microbial activities by altering microbial biomass^8, 9^ and bacterial^10, 11^ and fungal communities^12, 13^, and thereafter change their subsequent ecological function. In addition to N, crop rotations^14^, precipitation^15^, temperature^16^ and season^17^ strongly affect the composition, richness and diversity of soil microbial communities. Therefore, it is likely that research using single time-point sampling cannot differentiate the influences causing variation in microbial community structure. Understanding the bacterial community in response to N gradients and seasonal variations will help determine the ‘real’ effect of N and guide us in the appropriate development of agricultural ecological systems for food production.

Black soil, belonging to the pachic Haploborolls subtype of Haploborolls in the Boroll suborder, is one of the most fertile soils in China, and the agricultural area with this soil is very important for grain production and cultivation^17^. Very high inputs of inorganic fertilizers in this region have seriously degraded the soil physicochemical properties and environmental health since the 1950s^18^. These nutrient-based alterations of microbiota are reflected in significant shifts in the soil N-cycling and wider communities, including denitrifying bacteria^18, 19^, ammonia-oxidizing bacteria^20^ and the whole bacterial kingdom^11^. Ramirez *et al*.^21^ observed consistent effects of N fertilization on microbial respiration regardless of soil and N fertilizer type. However, in black soils, whether such effects of N occur over two continuous crop seasons remains unclear.

Long-term fertilization trials in Heilongjiang Province, China, allow investigations of the effects of repeated additions of N fertilizer on soil microorganisms in black soil. In this experimental field, the influence of higher levels of N and phosphorus (P) fertilizer treatments on bacterial communities was greater than that of lower levels after wheat harvest in 2013 in our previous study^11^. However, soybean was cultivated in 2014 and the weather, including mean annual temperature and precipitation, differed from that in 2013. Meanwhile, the soil concentration of nitrate (NO_3_^−^) – shown to be an important factor in shaping bacterial communities after 34 years of N and P addition in our previous study^11^ significantly differed in the two crop seasons. In the present study, using experimental gradients of N, the specific questions addressed were as follows: (i) Could N additions have consistent effects on the abundance and composition of the entire soil bacterial kingdom and the dominant phyla/classes/genera in both crop seasons? (ii) Will shifts in specific bacterial taxa correspond with the fertilizer-induced changes in soil pH and/or NO_3_^−^ concentration? Thus, we used long-term N experiments to assess the influence of N additions on bacterial community abundance, composition and diversity using real-time PCR coupled with pyrosequencing targeting the bacterial 16S rRNA gene.

## Results

### Soil and crop responses

In the wheat season, the N fertilization significantly (*P* < 0.05) affected the soil properties and wheat yields. Soil dissolved inorganic N (NH_4_^+^ and NO_3_^−^) was generally correlated with the N gradient, in the ranges of 33.8–40.7 and 7.7–24.5 mg kg^−1^ soil, respectively, accompanied by a corresponding decrease in soil pH from 6.5 to 4.6. The N addition also induced significant increases in soil Avail P from 9.3 to 15.3 mg/g soil and OM from 27.3 to 29.5 g kg^−1^ soil. From low to high fertilization, wheat yield significantly increased from 1548 to 2155 kg ha^−1^ (Table S1).

In the soybean season, the three soil properties (pH, Avail P and NO_3_^−^) and soybean yield were also significantly correlated with the N fertilization regime (*P* < 0.05). For low and high fertilization, pH was 6.5 and 5.4, respectively, corresponding soil NO_3_^−^ content was 2.4 and 5.6 mg g^−1^ soil, and soil Avail P was 1.8 and 64.9 mg g^−1^ soil. From low to high fertilization, soybean yield significantly increased from 1800 to 2761 kg ha^−1^ (Table S1).

### Effects of long-term N inputs on bacterial 16S rDNA copy numbers

**Figure 1:**
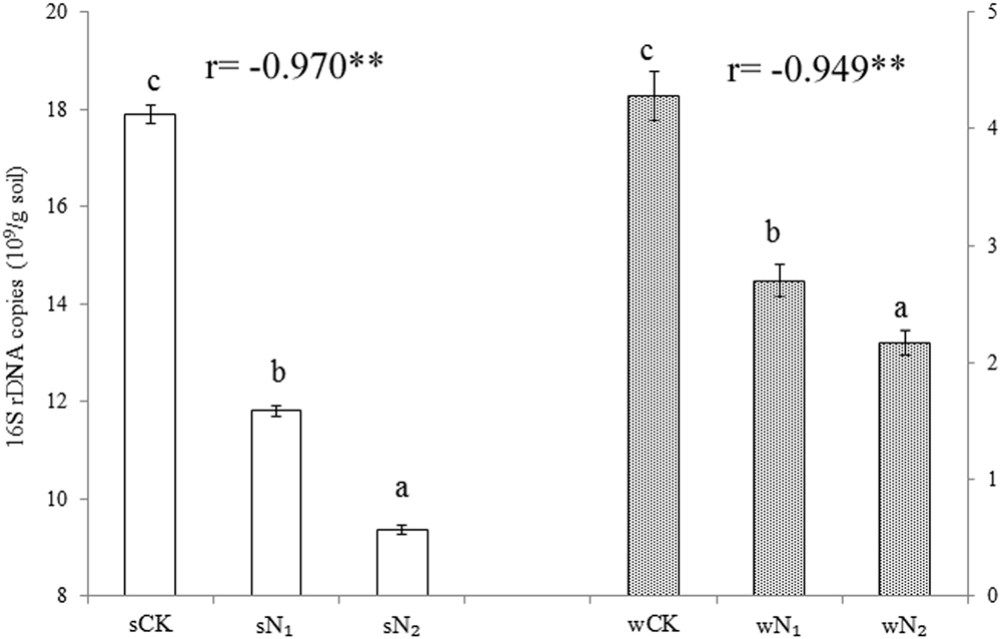
Abundance of bacteria as indicated by the numbers of 16S rDNA copies measured using quantitative PCR. sCK, sN_1_ and sN_2_ indicate different N treatments in the soybean season; wCK, wN_1_ and wN_2_ indicate different N treatments in the wheat season. The ‘r’ indicates Pearson’s correlation coefficient between N added and 16S rDNA copies (**P < 0.01, *P < 0.05).

The long-term N inputs significantly decreased the number of bacterial 16S rDNA copies (Fig. 1). These effects were significant (*P* < 0.01) on gene copy numbers/g soil in both wheat (r = −0.949) and soybean seasons (r = −0.970). For low and high N inputs, the numbers of bacterial 16S rDNA copies g^−1^ soil were 4.28 × 10^9^ and 2.17 × 10^9^ in the wheat season and 1.79 × 10^10^ and 9.35 × 10^9^ in the soybean season, respectively (Fig. 1).

### Differences in bacterial alpha diversity between different N fertilizer regimes

After OTU picking and chimera checking, a total of 2,078,821 high-quality sequences were assigned to 5148 non-singleton OTUs, resulting in the classification of 533 taxa at genus level. The numbers of OTUs in soil samples were in the range of 1624–2467 in the six fertilizer treatments (Table S2). The Good’s coverage values were in the range of 85.5–96.0% at 97% similarity cutoff, indicating sufficient sequence reads to capture the bacterial diversity in these soils (Table S2).

**Figure 2:**
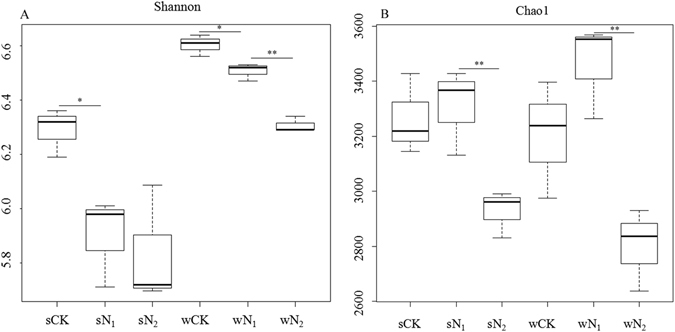
Differences in bacterial community diversity and richness between different N fertilizer regimes. (**A**) Community diversity between different N fertilizer regimes (both wheat and soybean seasons). (**B**) Community richness between different N fertilizer regimes (both wheat and soybean seasons). Asterisks show significant differences between samples (**P < 0.01, *P < 0.05, Tukey r-test). sCK, sN_1_ and sN_2_ indicate different N treatments in the soybean season; wCK, wN_1_ and wN_2_ indicate different N treatments in the wheat season.

Several alpha diversity measures were calculated, including estimated OTUs, Shannon’s diversity index (Fig. 2A) and Chao1 (Fig. 2B). For community Shannon diversity comparison, lower N addition samples (N_1_) had significantly lower Shannon values than those without N addition (CK) in both crop seasons (*P* < 0.05, Fig. 2A). Moreover, in the wheat season, the higher N addition (N_2_) led to significantly lower Shannon values than N_1_ samples did (*P* < 0.01, Fig. 2A). In the soybean season, Shannon values in N_2_ samples were lower than those in N_1_, although there was no significant difference between them. Shannon values in the two crop seasons were significantly negatively correlated with N fertilization inputs (*P* < 0.01, for both cases; Table S2).

There was no significant difference in community richness between N_1_ and CK samples, while N_2_ samples had significantly (*P* < 0.01) lower richness (Chao1) than N_1_ samples (both wheat and soybean seasons) (Fig. 2B).

### Differences in bacterial composition

**Figure 3:**
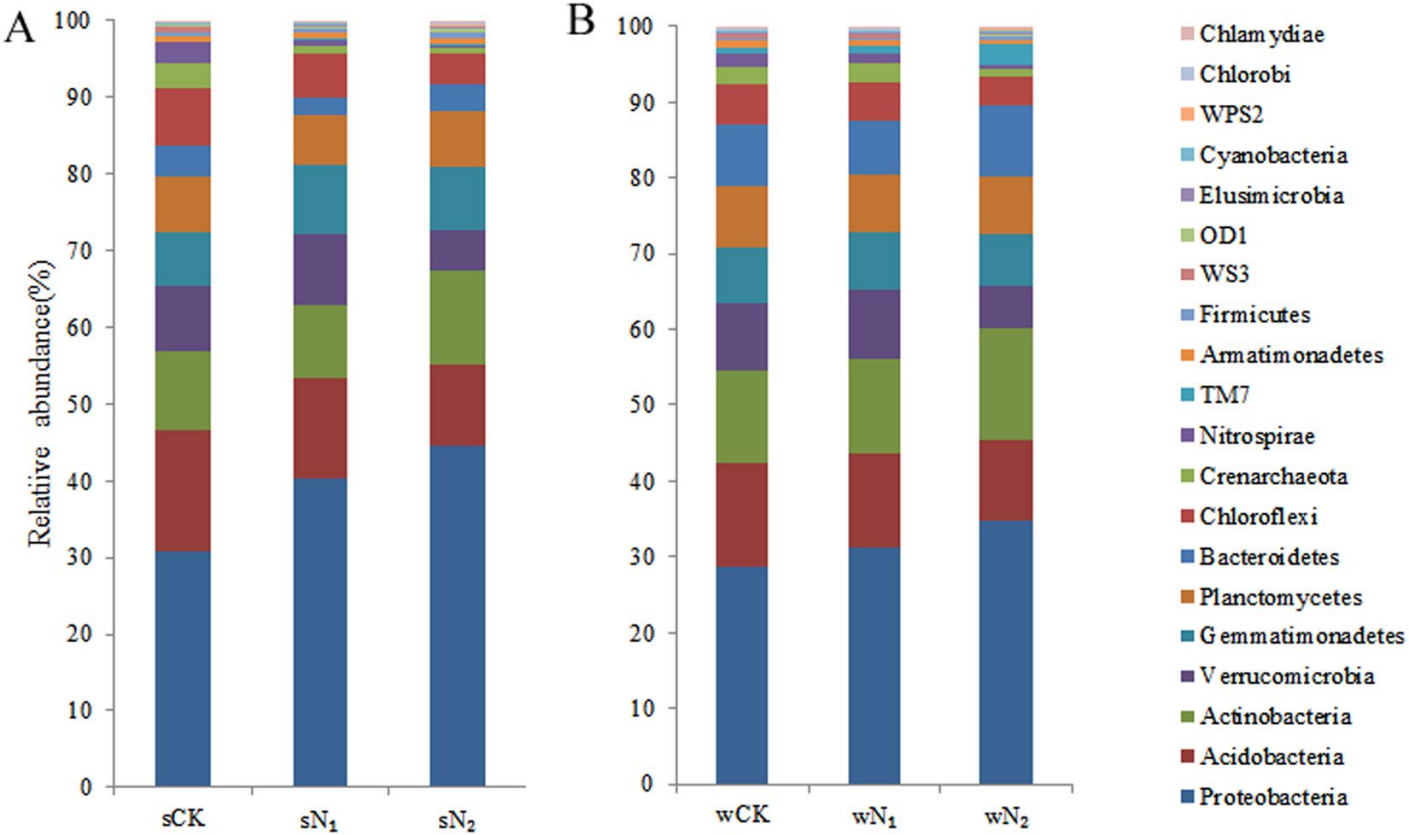
Bacterial compositions of different N fertilizer regimes. Each bar represents the average relative abundance of each bacterial taxon within a group. The top 20 abundant phyla are shown (relative abundance >0.01%). sCK, sN_1_ and sN_2_ indicate different N treatments in the soybean season; wCK, wN_1_ and wN_2_ indicate different N treatments in the wheat season.

**Figure 4:**
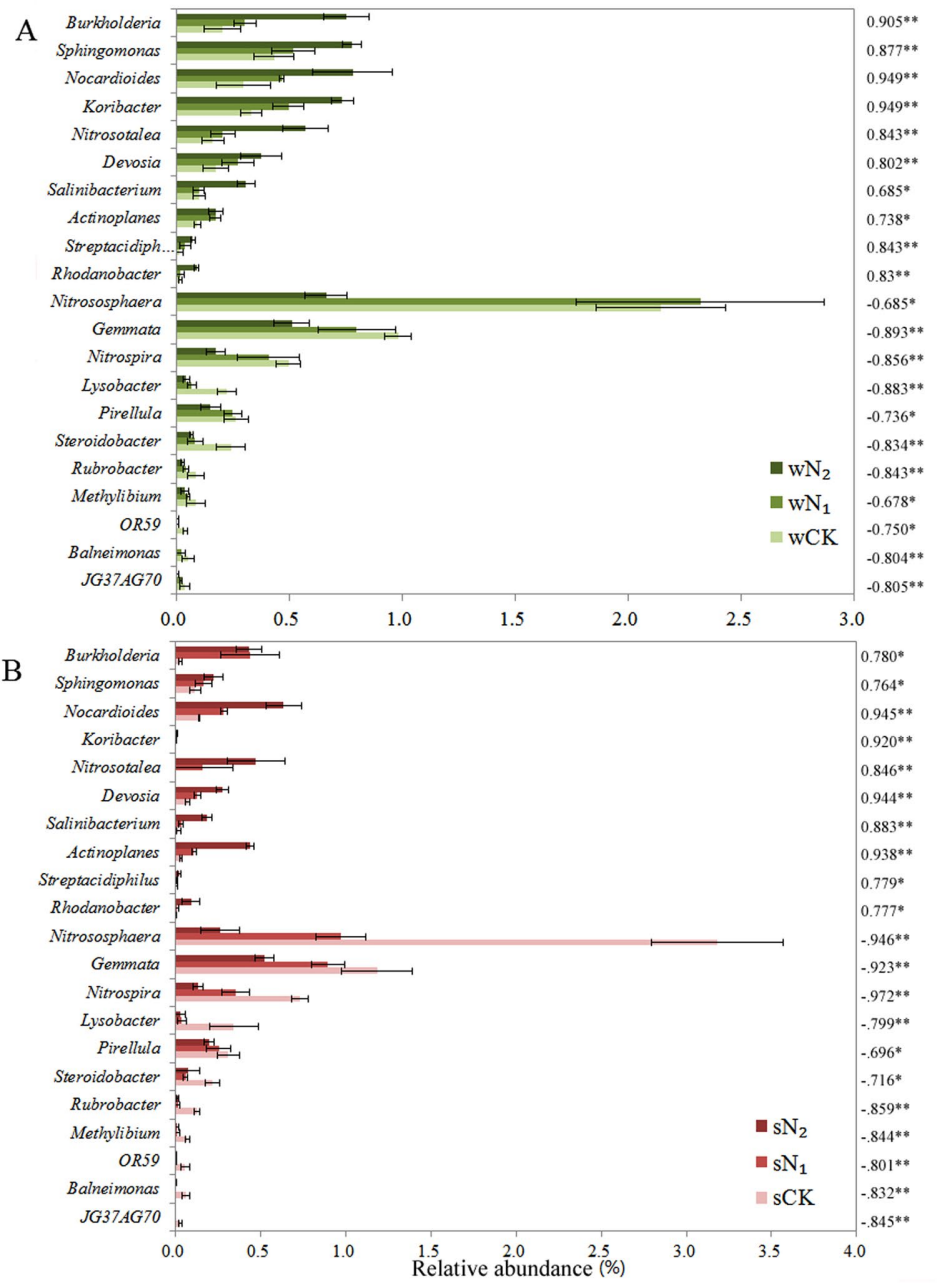
Changes in the relative abundances of bacterial genera across the N gradients in the wheat (**A**) and soybean (**B**) seasons. Only shown are those classes correlated with N added (Spearman’s r-values). Bars indicate one standard deviation. Asterisks show significant correlations (**P < 0.01, *P < 0.05). sCK, sN_1_ and sN_2_ indicate different N treatments in the soybean season; wCK, wN_1_ and wN_2_ indicate different N treatments in the wheat season.

Analysis of the community composition under different N fertilizer regimes in the two crop seasons showed that the top abundant 20 phyla accounted for 99.7% of the reads (Fig. 3). Proteobacteria was the most abundant phylum, representing 28.5–44.5% of the total sequences (Fig. S1). The bacterial communities in N_2_ samples differed from those in N_1_ and CK samples by showing higher relative abundances of Proteobacteria in both wheat and soybean seasons (Figs 3 and S1). Classes Alphaproteobacteria, Gammaproteobacteria and Deltaproteobacteria occupied 47.9, 13.0 and 11.7% of the sequences of this phylum, respectively (data not shown). With increasing N, the relative abundance of Alphaproteobacteria and Gammaproteobacteria increased significantly, and were highly positively correlated with N addition in wheat and soybean seasons (r = 0.949, *P* < 0.01, for both cases; Fig. S2A and B). However, the relative abundance of Deltaproteobacteria decreased significantly with increasing N for wheat (r = −0.949, *P* < 0.01) and soybean seasons (r = −0.738, *P* < 0.01; Fig. S2E). In the Alphaproteobacteria class, genera *Devosia* and *Sphingomonas* were significantly positively correlated with N addition (r = 0.944 and 0.764, respectively; *P* < 0.05), whereas *Balneimonas* showed the opposite trend (r = −0.832, *P* < 0.01; Fig. 4).

Acidobacteria was the second most abundant phylum in the sampled soils, and constituted 10.5–15.6% of total sequences (Fig. 3). The N addition significantly (*P* < 0.01) decreased the relative abundance of this phylum in both seasons (Fig. S1). Class Solibacteres occupied 19.4% of sequences of this phylum and was positively correlated with N addition in wheat (r = 0.843, *P* < 0.01) and soybean seasons (r = 0.896, *P* < 0.01; Fig. S2D). Classes Chloracidobacteria and Acidobacteria occupied 33.8 and 17.5% of sequences of this phylum, respectively (data not shown) and were negatively correlated with N addition in both seasons (*P* < 0.01 for both; Fig. S2F and G). At genus level, N addition significantly (r = 0.920, *P* < 0.01) increased the relative abundance of *Koribacter* (Fig. 4).

Actinobacteria constituted only 9.5–14.8% of the total sequences (Tables S4 and S5) – N addition increased the abundance of this phylum only in the wheat season (r = 0.738, *P* < 0.05; Fig. 3B); and also increased class *Thermoleophilia* in wheat (r = 0.949, *P* < 0.01) in soybean season (r = 0.896, *P* < 0.01; Fig. S2C). Four genera, *Salinibacterium*, *Actinoplanes*, *Nocardioides* and *Streptacidiphilus*, of Actinobacteria showed distinct correlations with N addition in both seasons (Fig. 4B).

Verrucomicrobia was the fourth most abundant phylum with 5.4–9.4% of sequences in these soils. Compared to that in unfertilized samples, the relative abundance of Verrucomicrobia in N_1_ samples increased not significantly, whereas those in N_2_ samples decreased significantly in both seasons (Tables S4 and S5). There was a significant (*P* < 0.01) decline in abundance of Chloroflexi and Nitrospirae (Tables S4 and S5) from unfertilized to high N treatments in both seasons.

### OTU-level bacterial β-diversity analysis and redundancy analysis

**Figure 5:**
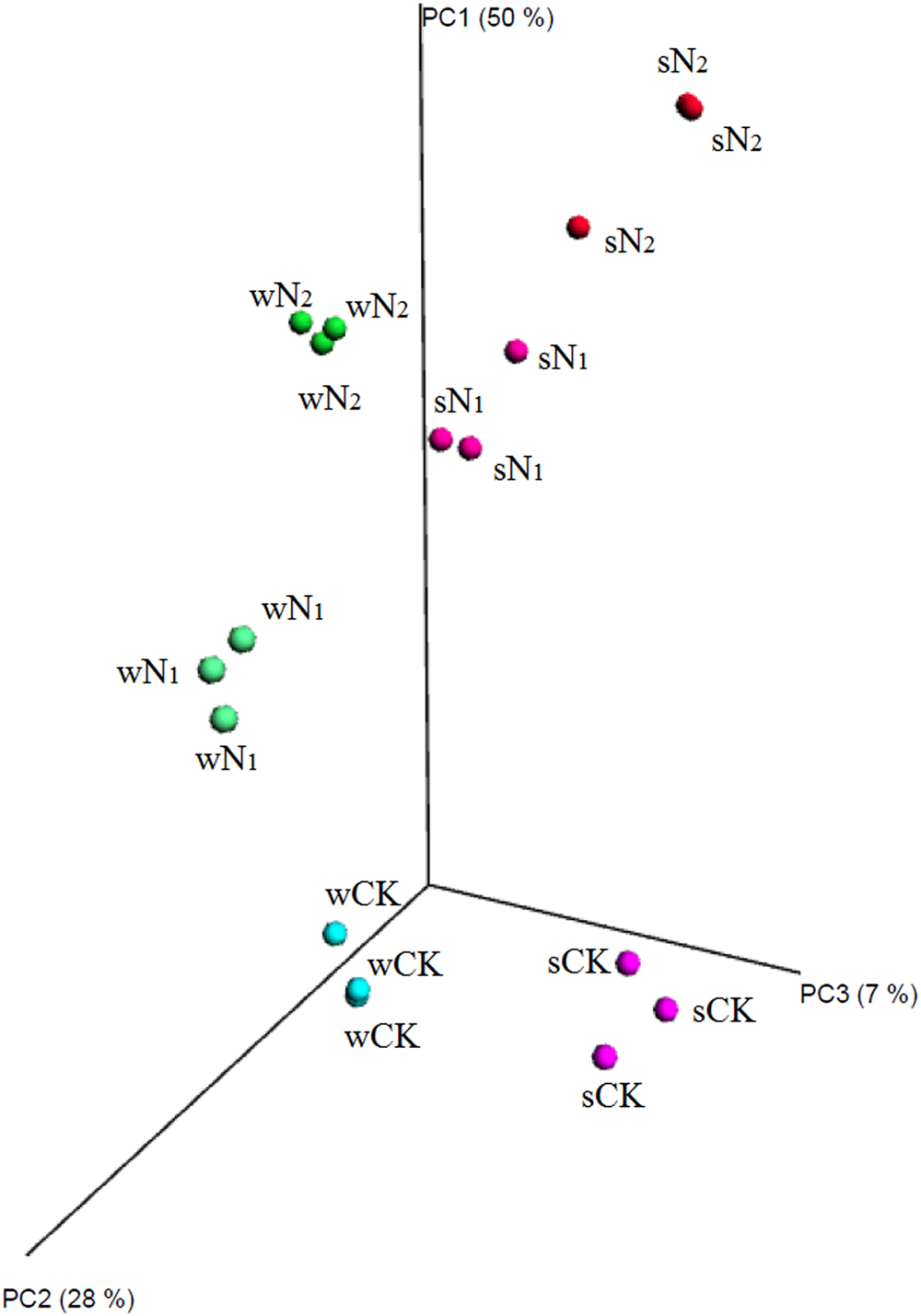
Principal coordinate analysis of pyrosequencing reads obtained from soils subjected to different fertilization regimes based on the weighted Fast UniFrac metric. The first three axes are shown and the percent of variance explained by each axis is given.

The PCoA based on the weighted UniFrac distance matrices (Fig. 5) clustered all three replicate soil samples within the same treatment plot together and they displayed stronger dissimilarities to those from other treatments. Across wheat and soybean seasons, the phylogenetic structure of the bacterial communities shifted in a similar manner. As N inputs increased, communities became progressively more distinct from those receiving no N fertilizer in both seasons. Furthermore, the overall weighted UniFrac distances between communities were highly correlated with N additions in both wheat (r = 0.932, *P* < 0.01) and soybean (r = 0.932, *P* < 0.01) seasons, as well as with edaphic characteristics (pH, NO_3_^−^ and Avail P; Table 1).

**Table 1:** Mantel test of UniFrac distances with soil properties and N addition in two seasons.

Crop season	N addition	pH	NO_3_^−^	NH_4_^+^	Avail P	Avail K	TN	OM
Wheat	0.932**	0.874**	0.506*	0.676**	0.688*	0.205	0.785**	0.365*
Soybean	0.932**	0.693*	0.489*	−0.029	0.475*	−0.076	0.277	0.222

Avail P indicates available phosphorus, Avail K is available potassium, TN is total N and OM is organic matter.

*P < 0.05; **P < 0.01.

According to the forward-selection option in CANOCO, soil pH (contribution of 35.8%, *P* = 0.002) NO_3_^−^ concentration (contribution of 30.4%, *P* = 0.002), Avail P (contribution of 13.8%, *P* = 0.002) and TN (contribution of 9.6%, *P* = 0.006) were the four most important contributors to the variation in bacterial communities (Table S3). These soil properties together explained 72.8% of the variation in microbial communities among samples. Based on this model, a total of 58.61% of the total variance was explained by the first two constrained axes of the RDA: the first axis explained 34.32% and the second 24.29% (Fig. S3).

## Discussion

### Variability in bacterial 16S rDNA copy numbers

In this study, N inputs significantly (*P* < 0.01) decreased 16S rDNA copy numbers in wheat (r = −0.949) and soybean seasons (r = −0.970), which partially addressed question concerning N addition consistently decreasing bacterial abundance. The result was in accordance with our previous research^11^, which concluded that bacterial 16S rDNA copy numbers declined remarkably across different doses of N and P fertilizer inputs. It was interesting that soil pH also showed the same trend with 16S rDNA copy numbers across N gradients in this experiment. Particularly, in N_2_ plots, after long-term urea-N fertilizer application, the soil was acidic and pH declined to 4.64 and 5.4 in the wheat and soybean seasons, respectively; the corresponding abundances of bacteria were 9.79 × 10^7^ and 5.92 × 10^7^ copies/g soil and declined by 49.2% and 47.8% compared to CK. The results indicated that bacterial strains had narrow pH optima and N fertilization induced a significant reduction in bacterial numbers^22^. A high correlation between the gradient of soil pH and the shift in 16S rDNA copy numbers was previously reported^23, 24^ and indicated that soil pH could have a major effect on population sizes of bacteria. As farmers use more inorganic N fertilizers, bacteria abundance will decrease with time – the fungi will grow faster than bacteria^25^ and this will lead to lower bacteria to fungi ratios in soil, which is an important parameter in assessing the balance of soil microbial communities^26^.

### Variability in bacterial community diversity

Recent studies have shown that overuse of inorganic N can reduce bacterial biodiversity^27^. In the present study, in both wheat and soybean seasons, there was a consistent decline in bacterial α-diversity (Shannon index) (Fig. 2A) across N gradients, and these shifts were driven largely by N addition (Table S2) – this was in good agreement with previous reports^27, 28^. The decline in biodiversity caused by N accumulation was reported to be due to stimulating the expansion of nitrophilous species and competitive exclusion of others^29^. Although soil pH^11^, soil type^30^, moisture^31^ and crop rotation^32^ have all been shown to be important factors affecting bacterial diversity, the direct or indirect interactions among plant, soils and bacterial diversity in response to N deposition remain unclear. However, in this study, the high correlation between N addition and bacterial diversity in two different crop seasons indicates that bacterial diversity may be affected more by N enrichment than crops. The loss of microbial diversity can alter terrestrial ecosystem processes, and suggests that the importance of functional redundancy in soil microbial communities has been overstated^33^. In the present study, a lower bacterial α - diversity in high N inputs soils may lead to a less stable ecosystem^34^, and in this sense very high N addition is not suitable for sustainable microbial functions and processes.

ACE and Chao1, two estimators of richness, revealed higher bacterial richness in control and N_1_ plots than that in N_2_ plots. However, their shifts were not related to N addition, and the result was similar to the report of Zhang *et al*.^35^.

### Variability in the composition of the dominant taxa

In both seasons, we observed consistent shifts in bacterial community composition (Fig. 3) and these shifts were driven largely by changes in the relative abundance of specific bacterial groups (Figs 3, S1 and S2), which addressed question concerning whether N additions consistently changed the bacterial community composition. Proteobacteria became more dominant in N_2_ treatments (34.71% and 44.47% in wheat and soybean seasons, respectively; Fig. 3) than in CK. Among them, the relative abundance of class Alphaproteobacteria increased by 33.6% and 77.6% compared to that in CK in wheat and soybean seasons, respectively (Fig. 3B). Alphaproteobacteria (e.g. family Acetobacteraceae) can use recalcitrant forms of C and supply important C intermediates to other microorganisms in acidic fermentation reactions^36^. There was obviously lower pH and higher Alphaproteobacteria abundance in N_2_ soils. *Devosia* species are N-fixing bacteria and have been shown to be positively correlated with potato yield^37^; and bacteria of genus *Sphingomonas* can metabolize C1 compounds^38^. An increase in these two genera in N_2_ soils may be an advantage of using urea fertilizers because these groups are also known to contribute to plant health by promoting plant growth and protecting against plant diseases^37, 39^.

Gammaproteobacteria abundance increased by 68.2% and 65.9% compared to the unfertilized controls in wheat and soybean seasons, respectively. Previous work also suggested that Gammaproteobacteria were more abundant in long-term N fertilized soils compared with unfertilized controls^35, 36^. Some Gammaproteobacteria species are considered to be organotrophic and can flourish in soils with large amounts of nutrients, and utilize reduced inorganic compounds such as ammonia, nitrite or NO_3_^−^ as energy sources^24^. For this reason, higher relative abundance of Gammaproteobacteria was observed in higher N addition treatments with higher concentrations of nitrite, NO_3_^−^ and OM. In contrast, Deltaproteobacteria abundance decreased across N gradients, and the decline patterns were consistent with response of Deltaproteobacteria to soils amended with inorganic N^4^.

Phylum Actinobacteria demonstrated a positive trend with increasing N addition in both seasons (Fig. S1), which was not in accordance with previous results showing that Actinobacteria decreased at low pH^40^. However, this shift was consistent with the prediction of Ramirez *et al*.^41^ that Actinobacteria were copiotrophic groups with fast growth rates, relying on more labile C sources and more likely to increase in abundance with increased nutrient inputs^28^, such as in high N amendments (N_2_) in the present study. In contrast, the relative abundances of Acidobacteria and Verrucomicrobia decreased across N gradients in both seasons, possibly because they are oligotrophic groups^42^ with slower growth rates, and in all likelihood, with the ability to metabolize nutrient-poor and recalcitrant C substrates. This shift is consistent with the microbial N mining hypothesis that soil microbes reduce decomposition of recalcitrant C and lead to a shift towards labile C decomposition under N-enriched conditions^43^. Thus, they have lower competitiveness than copiotrophic microbes in nutrient-rich soil. The presumed taxon-specific responses might be an indirect N effect arising from the increase in organic C availability associated with the increase in wheat and soybean productivity in soils amended with N. The bacterial taxon responses observed across the N gradients are consistent with characteristics of metagenomes^44^, suggesting that the communities became more copiotrophic as N inputs increased.

### Factors driving the shifts in the bacterial community

Mantel tests showed that N addition was strongly related to the overall UniFrac distances between communities. This result highly corresponded to previous studies demonstrating that shifts in community composition were highly correlated with N additions in two long-term experimental N-gradient grasslands^21^. N fertilization decreased soil pH, and soil pH was highly correlated with UniFrac distance between bacterial communities. The RDA revealed that a large percentage (35.8%) of the variation within the pyrosequencing data was attributable to soil pH. The results validated question (ii) and previous work^11, 45^ also concluded that soil pH had a strong influence on soil bacterial community structure and diversity in both seasons. Meanwhile, concentration of NO_3_^−^ in soil (contribution of 30.4%, r = 0.506) was also an important factor driving the shift of bacterial community according to the Mantel test (Table 1) and RDA (Table S3) results, which was consistent with our previous studies^11^ and addressed question (ii). Furthermore, soil Avail P (contribution of 13.8%, r = 0.688, *P* = 0.002) was also significantly correlated with the variation in bacterial communities. Similar results were previously reported, in which both the bacterial^46^ and AM fungal communities^47^ were significantly correlated with soil Avail P^48^. Our results showed that long-term N addition in the absence of P had a significant effect on the soil Avail P content, making it a limiting nutrient for some sensitive bacterial groups. For example, the relative abundances of order Legionellales (in class Gammaproteobacteria) and Sphingomonadales (in class Sphingobacteria) were highly positively correlated with the Avail P concentration in our previous study^11^.

Although soybean and wheat can produce different root exudates, and root exudates shape soil bacterial community structure^49^, we observed consistent shifts in bacterial abundance, diversity and community structure in both wheat and soybean seasons. These shifts were driven largely by changes in the dose of N addition. However, our observed trends were in direct contrast with predictions, suggesting that crop root exudates may be important in structuring the communities, but were not the dominant factor responsible for the pronounced shifts in bacterial community composition across these N gradients. The bacterial compositional changes caused by N fertilizer were more dramatic than those caused by crop root exudates in our experiment.

## Conclusion

By simultaneously investigating bacterial community responses to urea -N across two contrasting crop seasons, we determined that N additions enhanced the concentration of soil N nutrients, but decreased bacterial 16S rDNA copy numbers. Our work suggests that N inputs elicited similar responses in the entire soil bacterial community structure and the dominant groups, although the temperature, precipitation and cultivated crops significantly differed in the two continuous crop seasons. Moreover, we observed a significant and distinct shift in the bacterial community resulting from N fertilizer regimes and their subsequent effect on edaphic characteristics, while crop season also led to a significant but much smaller variation in bacterial community structure. This work highlights that shifts of bacterial community may be important in understanding the corresponding consequences of urea-N addition and provides an important step towards identifying the likely community shifts associated with urea-N addition.

## Methods

### Field description and soil sampling

The experimental field is located in Harbin city, Heilongjiang Province, China (45°40′ N, 126°35′ E and altitude 151 m). This region has a temperate continental monsoon climate, with a frost-free period of 135 d. Wheat, soybean and maize were continuously grown on the field and the fertilization experiment was commenced in 1980, including three replicates (each plot was 9 m × 4 m) in a randomized complete block design. In the present study, we analyzed soil samples from three treatments. In early July annually since 1980, each plot in each row was randomly assigned to one of three fertilizer treatments: CK (without fertilizer), N_1_ (150 kg urea ha^−1^ y^−1^) and N_2_ (300 kg urea ha^−1^ y^−1^). The period of crop growing started in early April and ended in late September every year and we collected soil samples in September 2013 and 2014 after wheat and soybean harvests, respectively. The annual average soil temperature, 10 cm below the surface of soil was 7.4 and 5.9 °C, and annual precipitation was 2262 and 296 mm in 2013 and 2014, respectively (http://cdc.nmic.cn/home.do). Ten cores (7.5 cm in diameter) were randomly collected from the plow layer of soil (5–20 cm) in selected subplots within each treatment plot. The cores from each replicate plot were mixed together, and transported to the laboratory on ice. Samples were sieved (2 mm pore size) to remove roots, debris and stones, and divided into two parts: one was stored at −80 °C for molecular analysis, and the other was air-dried and used to determine soil properties.

### Soil physicochemical properties and crop yield

Soil pH was measured using a glass electrode meter in a soil water ratio (mass: volume) of 1:1, after being shaken for 1 h^50^. Organic matter (OM) was determined using the potassium dichromate external heating method^51^. Total N (TN) was measured according to Strickland and Sollins^52^. Soil KCl-extractable NO_3_^−^ and ammonium (NH_4_^+^) were quantified by extraction with 2 M KCl, steam distillation and titration^53^. Available P was extracted with sodium bicarbonate and then determined using the molybdenum blue method^54^. Available K was extracted with ammonium acetate and determined by flame photometry^22^. Wheat and soybean yield were determined by the soil testing laboratory in Harbin at the black soil experimental station of the Heilongjiang Academy of Agricultural Sciences.

### Real-time PCR

Soil (0.25 g) was used for DNA extraction using the PowerSoil DNA extraction kit (MOBIO Laboratories Inc., Carlsbad, CA, USA) following the manufacturer’s method, modified with an additional incubation step at 65 °C for 10 min followed by 2 min of bead beating^55^. To minimize the DNA extraction bias, three successive extractions of microbial DNA from the same soil samples were combined and purified using a DNeasy Tissue kit (Qiagen, Valencia, CA, USA)^2^. Quantitative real-time PCR was performed to determine the relative 16S rRNA gene abundance. We used the 515f/806r primer sets^56^ to quantify the total bacterial populations. The standard templates were made from 10-fold dilutions of linearized plasmids containing the gene fragment of interest that was cloned from amplified pure 16S rRNA gene. The *R* ^*2*^ of the standard curve was >0.99.

### Sequencing of 16S rRNA gene

DNA was amplified using the 515f/806r primer set with the reverse primer containing a 6-bp error-correcting barcode unique to each sample. Three replicate PCR reactions for each sample were purified, and pyrosequencing was conducted on an Illumina Miseq 2 × 250 platform according to the protocols of Caporaso *et al*.^56^. Raw sequences were deposited in the NCBI Sequence Read Archive with accession number SRX1126010.

### Bioinformatics

The quality filtering of raw reads and operational taxonomic unit (OTU) clustering was done with mothur v1.32^57^. The quality filter removed sequences outside the range of 200–300 bases long and those with primer mismatches. OTUs were picked using a de novo OTU picking protocol with a 97% similarity threshold. Rare OTUs (<4 reads each; <2% of total reads) were excluded from this analysis in order to reduce the datasets and thus provide more consistent alignments. High-quality sequences were classified using the Greengene database.

Alpha diversity analysis included Shannon index, Chao1 and number of OTUs within each sample was calculated in the QIIME pipeline, based on rarefaction of the OTU table. To compare between-sample differences in the total bacterial community composition, unweighted and weighted UniFrac distances^58^ were calculated with the QIIME pipeline^55^. Principal Coordinate Analysis (PCoA) was performed on the basis of the weighted UniFrac distance measured, and coordinates were used to draw 3D graphical outputs^57^.

### Statistical analysis

The concentrations of OM, TN, NO_3_^−^, NH_4_^+^ and available P and pH in soil samples were tested using a one-way analysis of variance (ANOVA). Correlations between soil properties, the relative abundances of each bacterial group, and estimated diversity levels with N inputs were tested for significance using Pearson’s correlations in the SPSS (v19.1) statistical package. To analyze changes in phylogenetic structure, pairwise weighted UniFrac distance matrices were compared against N addition and each of the measured soil properties using Mantel tests (999 permutations) in the R statistical language (x64 3.1.3) using the ‘vegan’ package following methods of Ramirez *et al*.^36^. Redundancy analysis (RDA) was applied to visualize the effect of edaphic factors on bacterial community structure, and was carried out with CANOCO software (version 5.0, Microcomputer Power Inc., Ithaca, NY, USA) followed by Monte Carlo permutation tests (999 permutations).

## Electronic supplementary material

Supplementary Information
