# Supplementary material for: Consistent effects of nitrogen fertilization on soil bacterial communities in black soils for two crop seasons in China
Source: Sci Rep. 2017 Jun 12;7:3267. doi: 10.1038/s41598-017-03539-6 (PMC5468298; doi:10.1038/s41598-017-03539-6)
Supplement: Supplementary file 1 — Supplementary Information [file 41598_2017_3539_MOESM1_ESM.doc]

## Title: Consistent effects of nitrogen fertilization on soil bacterial communities in black soils for two crop seasons in China

Jing Zhou^a,b^, Xin Jiang^a,d*^, Dan Wei^c^, Baisuo Zhao^d^, Mingchao Ma^a,d^, Sanfeng Chen^b^, Fengming Cao^a,d^, Delong Shen^d^, Dawei Guan^a^ and Jun Li^a,d*^

^a^Institute of Agricultural Resources and Regional Planning，Chinese Academy of Agricultural Sciences，Beijing 100081, PR China

^b^College of Biological Sciences, China Agricultural University, Beijing, 100094, PR China

^c^The Institute of Soil Fertility and Environmental Sources, Heilongjiang Academy of Agricultural Sciences, Harbin, 150086, PR China

^d^ Laboratory of Quality＆Safety Risk Assessment for Microbial Products (Beijing), Ministry of Agriculture*,* Beijing 100081, PR China

*Corresponding author: Jun Li and Xin Jiang

E-mail: [lijun01@caas.cn](mailto:lijun01@caas.cn) and jiangxin@caas.cn.

Tel: +86-10-82106208.

FAX: +86-10-82108702

**
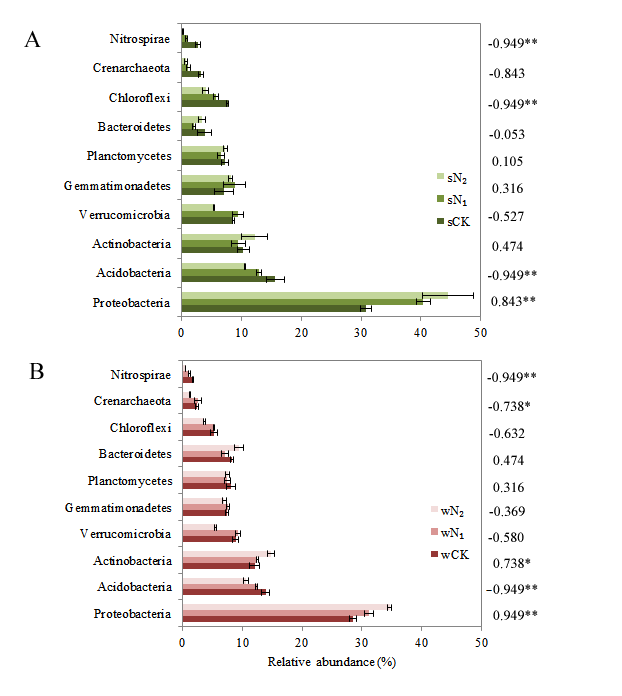
**

**Figure S1.** Changes in the relative abundances of bacterial taxa (at phylum level) across the N gradients in the soybean(A) and wheat (B) seasons. Only those classes with average relative abundances > 1% for either of the seasons are shown. The numbers on the right indicate the correlation (Spearman’s r-values) between N added and relative abundances. Bars indicate one standard deviation. Asterisks show significant correlations (** *P*<0.01, * *P*<0.05).sCK, sN_1_ and sN_2_indicate different N treatments in the soybean season; wCK, wN_1_ and wN_2_ indicate different N treatments in the wheat season.


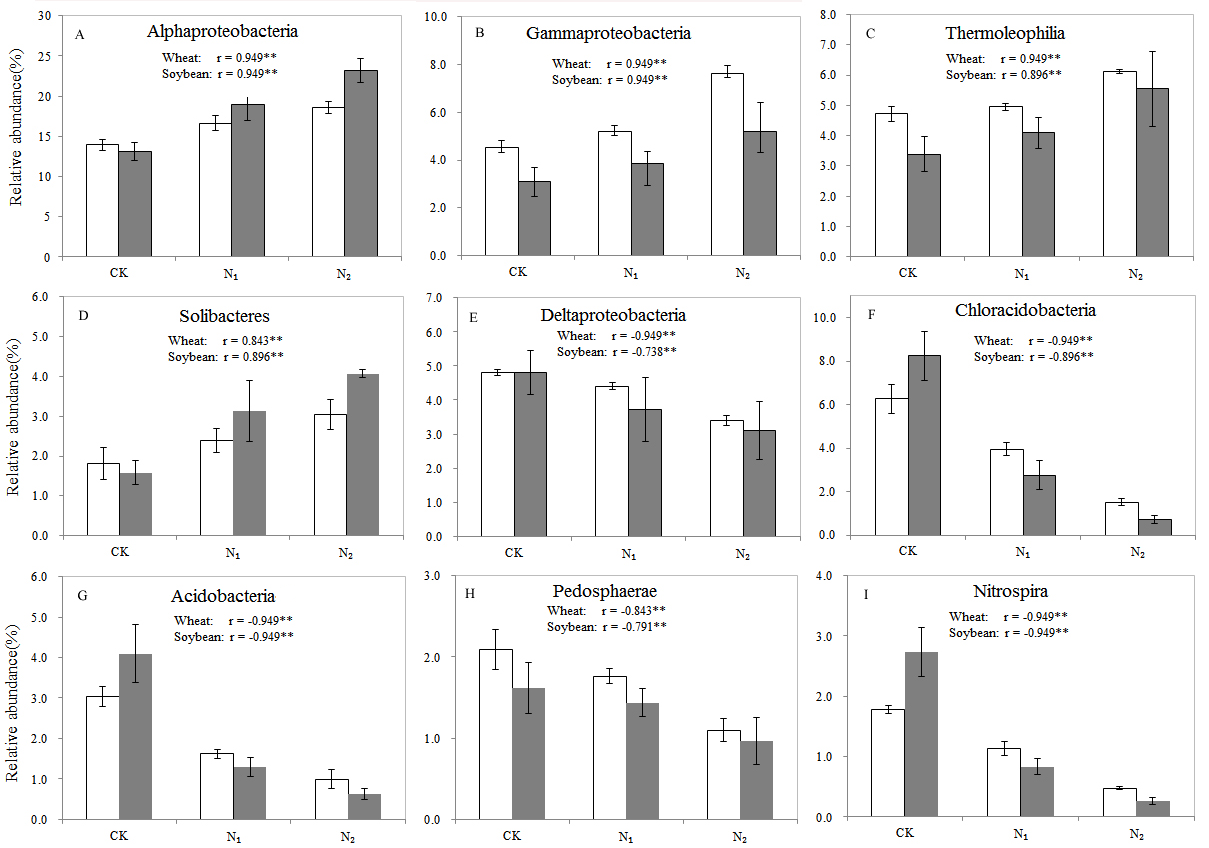


**Figure S2.** Changes in the relative abundances (>1%) of specific bacterial taxa (at class level) across the N gradients, asdetermined by Pearson’s correlationcoefficient (r). Seasons: whitebars, wheat; graybars, soybean.


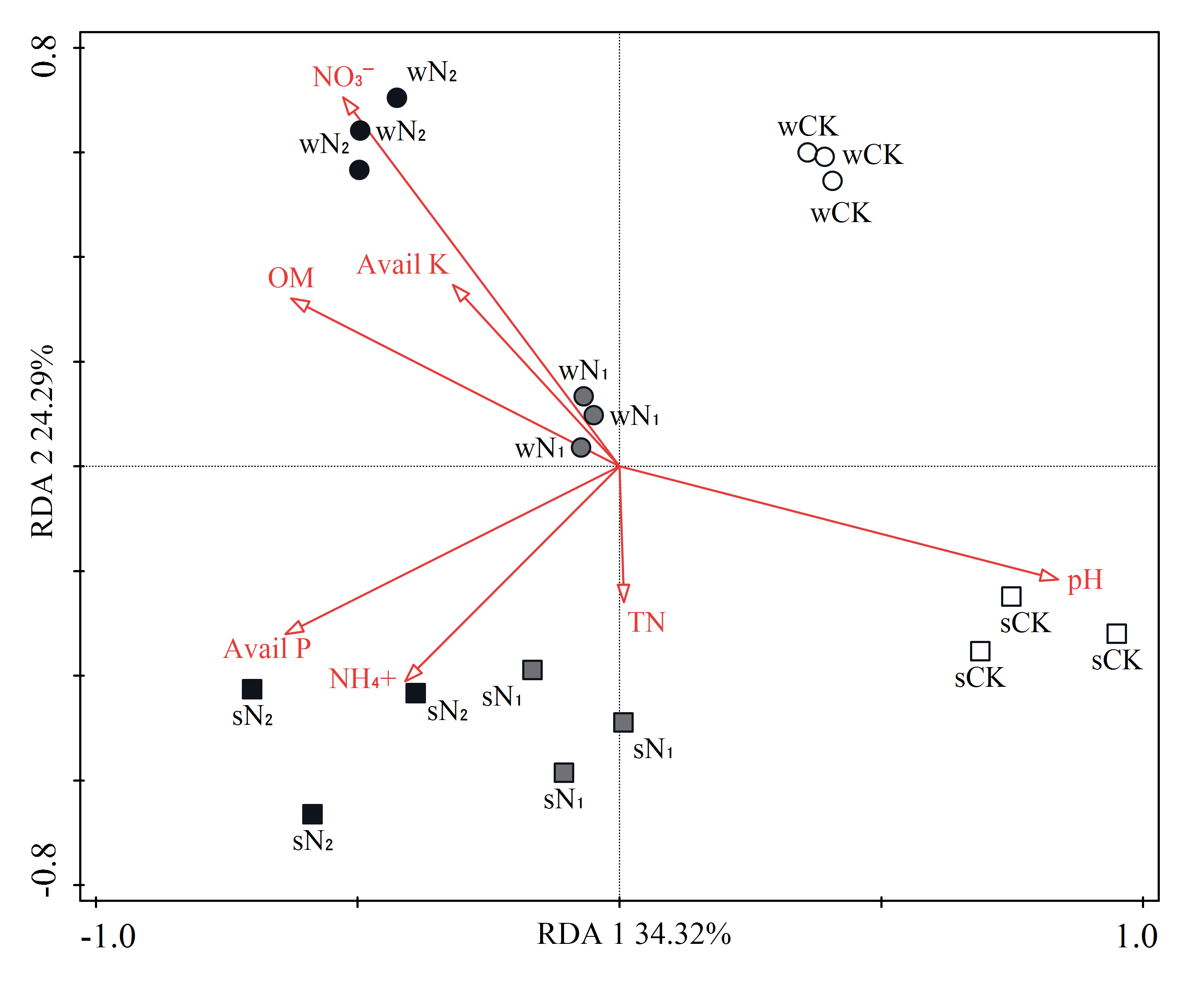


**Figure S3.** Redundancy analysis of soil bacterial communities and soil characteristics for individual samples. Soil factors indicated in red text include AvailP (available phosphorus), pH, NH_4_^+^ (soil concentration of NH_4_^+^), NO_3_^–^ (soil concentration of NO_3_^–^), TN (total nitrogen) and OM (organic matter). Shapes denote seasons: squares, soybean; circles, wheat. Colors denote N treatments: no N added (CK), white; low N (N_1_), gray; high N (N_2_),

Table S1

Soil properties and crop yields of different fertilizer samples in wheat and soybean seasons.

| Crop season | Treatments | pH | NO_3_^–^  (mg kg^–1^) | NH_4_^+^  (mg kg^–1^) | Avail P  (mg kg^–1^) | Avail K  (mg kg^–1^) | TN  (g kg^–1^) | Organic Matter  (g kg^–1^) | Crop yield  (kg ha^–1^) |
| --- | --- | --- | --- | --- | --- | --- | --- | --- | --- |
| Wheat | CK | 6.4±0.02c | 7.7±0.06a | 33.8±1.1a | 9.3±0.31a | 176.6±7.88a | 1.2±0.01a | 27.3±0.43a | 1548±280a |
|  | N_1_ | 5.6±0.02b | 8.5±0.06b | 36.9±2.51a | 10.9±0.64b | 183.2±20.47a | 1.3±0.01b | 28.3±0.92ab | 2140±230b |
|  | N_2_ | 4.6±0.02a | 24.5±0.12c | 40.7±1.16b | 15.3±0.31c | 216.6±39.93a | 1.4±0.03c | 29.5±0.99b | 2155±248b |
|  | Correlation^a^ | –0.995** | 0.886** | 0.894** | 0.956** | 0.589 | 0.973** | 0.801** | 0.707* |
| Soybean | CK | 6.5±0.06b | 2.4±1.02a | 34.8±0.57a | 1.8±0.22a | 178±7.16a | 1.5±0.02b | 25.7±2.72a | 1800±167a |
|  | N_1_ | 5.6±0.12a | 5.1±0.45ab | 48.4±11.68a | 3.8±0.67a | 185.1±4.5a | 1.3±0.08a | 27.9±0.75a | 2225±217b |
|  | N_2_ | 5.4±0.28a | 5.6±1.68b | 41.7±9.32a | 64.9±5.51b | 174.3±5.9a | 1.4±0.01ab | 27.8±0.96a | 2761±158c |
|  | Correlation^a^ | –0.753* | 0.775* | 0.312 | 0.877** | –0.224 | –0.413 | 0.486 | 0.933** |

Values are mean ± standard deviation (*N* = 3). Values within the same column followed by different letters indicate significant difference (*P* < 0.05).

Avail P indicates available phosphorus, Avail K is available potassium, TN is total N and OM is organic matter.

Fertilizer regimes as described in Table 1

^a^Correlation indicates the Pearson’s correlation coefficient (r) between N added and soil properties (** *P* < 0.01, * *P* < 0.05).

Table S2

Effects of fertilizer regime on the OTUs, coverage, richness and diversity.

| Crop season | Treatments | OTUs^1^ | Coverage^2^ | Richness^3^ | | Diversity^4^ | |
| --- | --- | --- | --- | --- | --- | --- | --- |
|  |  |  |  | Chao1 | Ace | Shannon | Simpson |
| Wheat | CK | 1872±97ab | 0.855±0.004a | 3204±213b | 4421±300b | 6.61±0.042c | 0.005±0.001a |
|  | N_1_ | 2093±194b | 0.886±0.017a | 3132±389b | 4205±505b | 6.41±0.111b | 0.006±0.001b |
|  | N_2_ | 1624±164a | 0.899±0.023a | 2919±179a | 3813±173a | 6.3±0.039a | 0.006±0.001b |
|  | Correlation^5^ | –0.474 | 0.201 | –0.527 | –0.58 | –0.949** | 0.58 |
| Soybean | CK | 2467±916b | 0.955±0.007a | 3277±156ab | 3381±182a | 6.27±0.062b | 0.007±0.001a |
|  | N_1_ | 2299±175ab | 0.957±0.008a | 3127±285b | 3338±355a | 5.92±0.108a | 0.012±0.002ab |
|  | N_2_ | 2094±195a | 0.96±0.012a | 3099±217a | 3304±291a | 6.04±0.303a | 0.012±0.005b |
|  | Correlation^5^ | –0.344 | 0.102 | –0.621 | –0.109 | –0.841** | 0.832 |

Values are mean ± standard deviation (*N* = 3). Values within the same column followed by the different letters indicate significant difference (*P* < 0.05).

^1^ OTUs: operational taxonomic units (97% similarity).

^2^ Coverage: Good’s non-parametric coverage estimator.

^3^ Based on Chao1 and abundance-based coverage estimator (ACE) richness indices.

^4^ Based on Shannon and Simpson diversity indices.

^5^Correlation indicates the Pearson’s correlation coefficient (r) (** *P* < 0.01, * *P* < 0.05).

Table S3

Forward selection results of redundancy analysis.

| **Name** | **Explains %** | **Contribution %** | **Pseudo-F** | **P-value** |
| --- | --- | --- | --- | --- |
| pH | 26.1 | 35.8 | 5.6 | 0.002 |
| NO_3_^–^ | 22.1 | 30.4 | 6.4 | 0.002 |
| Avail P | 10.1 | 13.8 | 3.4 | 0.002 |
| Total N | 7.0 | 9.6 | 2.6 | 0.006 |
| NH_4_^+^ | 3.0 | 4.2 | 1.1 | 0.354 |
| Avail K | 2.3 | 3.2 | 0.9 | 0.668 |
| OM | 2.2 | 3.1 | 0.8 | 0.646 |

Table S4

Effects of fertilizer regimes on the top 20 abundant phyla (relative abundance > 1%) in soybean season.

| **Phylum** | **sCK** | **sN_1_** | **sN_2_** |
| --- | --- | --- | --- |
| Proteobacteria | 30.83±0.9a | 40.43±1.18b | 44.47±4.26b |
| Acidobacteria | 15.64±1.48c | 12.95±0.46b | 10.57±0.05a |
| Actinobacteria | 10.33±1.04a | 9.48±1.23a | 12.23±2.20a |
| Verrucomicrobia | 8.67±0.18b | 9.38±0.92b | 5.40±0.13a |
| Gemmatimonadetes | 7.06±1.58a | 8.90±1.85a | 8.21±0.37a |
| Planctomycetes | 7.23±0.54a | 6.65±0.58a | 7.32±0.40a |
| Bacteroidetes | 3.84±1.19c | 2.12±0.25a | 3.43±0.57ab |
| Chloroflexi | 7.67±0.24c | 5.73±0.47b | 3.99±0.55a |
| Crenarchaeota | 3.25±0.43c | 1.13±0.33a | 0.74±0.28a |
| Nitrospirae | 2.74±0.41c | 0.83±0.14b | 0.26±0.06a |
| TM7 | 0.04±0.02a | 0.11±0.03a | 0.32±0.06b |
| Armatimonadetes | 0.80±0.05a | 0.85±0.12a | 0.77±0.07a |
| Firmicutes | 0.31±0.06a | 0.36±0.16ab | 0.76±0.33b |
| WS3 | 0.95±0.20c | 0.19±0.05b | 0.02±0.02a |
| OD1 | 0.09±0.03a | 0.23±0.05ab | 0.51±0.32b |
| Elusimicrobia | 0.12±0.05a | 0.22±0.03a | 0.28±0.16a |
| Cyanobacteria | 0.09±0.02a | 0.1±0.03a | 0.07±0.04a |
| WPS2 | 0.00±0.00a | 0.02±0.0a | 0.21±0.03b |
| Chlorobi | 0.09±0.03a | 0.06±0.03a | 0.11±0.09a |
| Chlamydiae | 0.01±0.01a | 0.05±0.03a | 0.06±0.04a |

Table S5

**Effects of fertilizer regimes on the top 20 abundant phyla (relative abundance > 1%) in wheat season.**

| **Phylum** | **wCK** | **wN_1_** | **wN_2_** |
| --- | --- | --- | --- |
| Proteobacteria | 28.58±0.61a | 31.2±0.77b | 34.71±0.35c |
| Acidobacteria | 13.9±0.63c | 12.37±0.23b | 10.62±0.41a |
| Actinobacteria | 12.05±0.82a | 12.48±0.17a | 14.77±0.57b |
| Verrucomicrobia | 8.85±0.53b | 9.27±0.39b | 5.52±0.14a |
| Gemmatimonadetes | 7.39±0.26a | 7.58±0.30a | 6.99±0.40a |
| Planctomycetes | 8.10±0.74a | 7.48±0.45a | 7.54±0.33a |
| Bacteroidetes | 8.23±0.26ab | 7.06±0.60a | 9.44±0.78b |
| Chloroflexi | 5.24±0.62b | 5.23±0.13b | 3.64±0.19a |
| Crenarchaeota | 2.38±0.31a | 2.55±0.63a | 1.25±0.12b |
| Nitrospirae | 1.78±0.07c | 1.14±0.12b | 0.48±0.03a |
| TM7 | 0.78±0.04a | 0.97±0.09a | 2.65±0.34b |
| Armatimonadetes | 0.84±0.07 | 0.92±0.14 | 0.67±0.02 |
| Firmicutes | 0.21±0.06a | 0.28±0.04a | 0.33±0.09a |
| WS3 | 0.58±0.16c | 0.27±0.04b | 0.14±0.09a |
| OD1 | 0.14±0.06a | 0.15±0.07a | 0.23±0.06a |
| Elusimicrobia | 0.19±0.05a | 0.22±0.04a | 0.21±0.06a |
| Cyanobacteria | 0.26±0.16a | 0.18±0.04a | 0.17±0.02a |
| WPS2 | 0.07±0.02a | 0.11±0a | 0.26±0.01b |
| Chlorobi | 0.1±0.03a | 0.11±0.01a | 0.08±0.05a |
| Chlamydiae | 0.03±0.02a | 0.08±0.03a | 0.07±0.02a |
